# Large‐scale proteomic and genomic analysis identify plasma proteins influencing human brain structure and Alzheimer’s disease risk

**DOI:** 10.1002/alz.094880

**Published:** 2025-01-09

**Authors:** Cyrus Ayubcha, Benjamin B Sun, Tian Ge, Chia‐Yen Chen

**Affiliations:** ^1^ Harvard Medical School, Boston, MA USA; ^2^ Harvard T.H. Chan School of Public Health, Boston, MA USA; ^3^ Biogen, Cambridge, MA USA; ^4^ Massachusetts General Hospital, Boston, MA USA; ^5^ Broad Institute of MIT and Harvard, Cambridge, MA USA

## Abstract

**Background:**

Recent advances in proteomics technology enable us to comprehensively examine the impact of the proteome on Alzheimer’s disease (AD) and intermediate phenotypes, such as brain imaging measures. Here, by leveraging large‐scale proteomic, brain imaging, and AD genome‐wide association studies (GWAS), we performed Mendelian randomization (MR) and colocalization analysis to investigate the impact of plasma protein levels on brain imaging measures and the link between plasma protein levels, brain imaging, and AD.

**Method:**

We leverage the protein quantitative trait loci (pQTL) datasets from the UK Biobank Pharma Proteomics Project (UKB‐PPP; 2,625 proteins by Olink assay) and deCODE genetics (Ferkingstad et al. 2021; 4,472 proteins by SomaLogic assay). UKB‐PPP and the deCODE study represent the largest plasma proteomics GWAS to date based on two different proteomics platforms, where the total sample sizes are over 30,000 for each. For the brain imaging and AD genetic datasets, we used the brain imaging GWAS from UKB (453 T1 MRI and DTI imaging; Smith et al. 2021), and AD GWAS from Bellenguez et al. 2022.

**Result:**

After extensive data processing and quality control, we performed two‐sample MR and identified significant associations in 170 protein‐DTI imaging pairs and 38 protein‐MRI pairs for 60 unique proteins from UKB‐PPP and 41 unique proteins from deCODE. Colocalization analysis further prioritized proteins related to AD implicated in previous studies. We further extended the association between plasma protein and brain imaging to AD by performing MR and colocalization analyses using pQTL and AD GWAS. As an example, we showed that plasma progranulin (P28799) level showed a significant effect on AD risk (*P* = 6.79×10^−12^), while supported by associations between plasma progranulin level and caudate volumes (right and left T1 DTI, *P* = 8.41×10^−6^).

**Conclusion:**

In summary, we presented the largest to date study for the impact of plasma protein levels on brain imaging measures and a novel approach to identify biomarkers for AD. Overall, the integration of large‐scale MRI, proteomics, AD phenotype, and genome‐wide data holds great promise for enhancing our understanding of AD, and ultimately facilitates biomarker discovery and therapeutic development.

